# 

**Published:** 1904-09-15

**Authors:** 


### ANNOUNCEMENTS.

The National Dental Association.—Because of the meeting of the Congress this body held no meeting this year except to elect officers and to transact such formal business as should come up for action. The next meeting will be held at Buffalo, N. Y. The following officers were elected: President, W. E. Boardman, of Boston; Vice-President for the East, J. I. Hart, New York; Vice-President for the South, L. P. Datterer, Charleston, S. C.; Vice-President for the West, Wm. Conrad, of St. Louis; Corresponding Secretary, C. S.

Butler, Buffalo, N. Y.; Recording Secretary, A. H. Peck, Chicago, Ills.; Treasurer, V. E. Turner, Raleigh, N. C. Drs. W. N. Cogan, C. S. Butler, and G. V. I. Brown were re-elected members of the Executive Committee. The former Executive Council was re-elected. The constitution was amended and hereafter the work of the sections will be done under three instead of six sections.

The Executive Council will fill the offices not elected.

---

The Ohio State Board held its semi-annual meeting July 28-30. Nineteen applicants were examined; twelve of them passed successfully.

